# Sugen, hypoxia and the lung circulation

**DOI:** 10.1177/20458940211051188

**Published:** 2021-10-04

**Authors:** Norbert F. Voelkel, Harm J. Bogaard

**Affiliations:** Department of Pulmonary Medicine, Amsterdam University Medical Centers, Amsterdam, the Netherlands; Cardiovascular Sciences, Vrije Universiteit Amsterdam, Amsterdam, the Netherlands

Twenty years ago, the paper was published that first described the Sugen Hypoxia rat model, a novel experimental model of pulmonary vascular disease and severe pulmonary arterial hypertension (PAH).^1^ With this short letter, we highlight some advantages and disadvantages inherent to the model and provide possible directions for future research. This piece is not intended as a comprehensive review of the model, but rather aims to celebrate two decades of work by many groups who have worked with an experimental model that was created by serendipity. We aim foremost to encourage all interested in the field to go back at the original paper and assess the road traveled since then.

The Sugen Hypoxia model first appeared in a paper published in the FASEB Journal,^1^ after the manuscript had been rejected by the major pulmonary journals. The rejection of a manuscript describing a novel model is not too surprising and should not encourage young investigators to give up on their work but urge them to continue to find ways to get their data published. At the time of this writing, the paper has been cited more than 800 times (in Google Scholar, while cited 295 times in PubMed) and the model is established in many laboratories around the world. With passage of time, many investigators have stopped referencing the original publication and this may indicate that the model has become common place, a standard model like the chronic hypoxia model and the monocrotaline model.

In reminiscing, it may be of more than historical interest to illustrate how contentious the early acceptance of this model of severe pulmonary arterial hypertension PAH model was. After all, it was based on a wrong hypothesis, and the team of investigators was caught by surprise when examining the lung histology for the first time, being confronted with an obliterative pulmonary vasculopathy^1^ that over time progresses to a plexogenic arteriopathy with striking resemblance to human PAH.^2^ The question asked most often by reviewers and conference attendees was: “You are treating the animals with an inhibitor of angiogenesis, and then you end up with angiogenesis?” Because chronic hypoxia, via hypoxia inducible factor (HIF)-1alpha, increased the expression of vascular endothelial growth factor (VEGF) in the rat lungs and VEGF was also highly expressed in the plexiform lesions in the lung tissue from patients with idiopathic PAH,^3^ the hypothesis was that the VEGF receptor blocker Sugen 5416 would inhibit chronic hypoxic pulmonary hypertension. That did not happen and the investigators needed to explain why severe angioproliferative PAH developed.

The Sugen/hypoxia model of angioobliterative PAH was not the first rodent model that had produced obliterative pulmonary vascular lesions. The pneumonectomy-monocrotaline model^4^ did not gain wide acceptance, likely because of the technical problems with the surgery and also because it did not provide a new hypothesis. The Sugen/hypoxia model generated interest because the first publication had identified the lumen-obliterating cells as endothelial cells and had documented that initial (early) apoptosis was necessary for the pathogenesis, because treatment with a pan-caspase inhibitor prevented disease development. Without initial apoptosis no subsequent endothelial cell growth.^5^ Follow-up publications provided evidence that the Sugen/hypoxia induced disease was remarkably restricted to the lung circulation, that targeted therapy had no influence on the pulmonary vascular lesions and that the number of obliterated small lung vessels predicted the level of right ventricular systolic pressure elevation.^6^,^7^ With passage of time the rat model was further explored, and one important question was: must in this two hit model the VEGF receptor blocker be paired with hypoxia as an obligatory second hit? This answer was soon provided as it was shown that instead of hypoxia, pneumonectomy^8^ and immune insufficiency in the athymic rat^9^ could substitute for hypoxia. Reviewers had frequently questioned whether therapies targeting VEGF signaling in human patients would affect the lung circulation. More recently, data on cancer patients treated with the VEGF receptor antibody bevacizumab provided some clinical context for the “angiogenesis paradox” of PAH in the Sugen PAH models,^10^ as it was shown that treatment of cancer patients with bevacizumab can induce proliferative pulmonary vascular changes.^11^

The Sugen/hypoxia model also allows to study right heart failure and its relationship with myocardial capillary rarefaction.^12^ The authors of the 2001 publication were concerned whether indeed the pathobiology which leads to severe PAH in this model could be solely explained by inhibited VEGF signaling. “Whether the action of Sugen 5416 is exclusively via VEGFR-2 blockade or via other effects is of great importance for the interpretation of our data and the mechanisms involved in the development of pulmonary hypertension.”

One component of the model highlighted more recently is the high lung tissue expression of genes encoding proteins of the arylhydrocarbon receptor (AhR)-cytochrome P450 axis.^13^ See also Masaki et al.^14^ and Dean et al.^15^ for further reading on the role of the AhR in PAH. This aspect of the model continues to be generally overlooked but needs to be considered as a potentially important contributor to the vascular cell proliferation and cell phenotype changes which characterize this model. In addition, Sitapara et al. reported that Sugen 5416 affects BMPRII signaling,^16^ providing yet another possible explanation for the development of PAH like vascular lesions after the double challenge of Sugen and hypoxia.

Now, 20 years later, investigators are still divided: some are critical and believe that the model does not represent all of the important features of the severe forms of human PAH,^17^ others continue to work with the model as a disease-relevant model for the preclinical testing of novel therapies.^18^ Today, as at the time of its first publication,^1^ the Sugen-hypoxia model is still only partially understood and remains controversial. Funding agencies and journal reviewers have criticized a lack of mechanistic insight that comes with experimental rat models. Many have insisted that the future of pulmonary vascular research lies in studying genetically engineered mice? Apropos, the mouse. It is important to note that treatment of mice with Sugen 5416 does not recapitulate the lung pathology observed in rats, perhaps because the two rodent species do not share the same repertoire of cytochrome P450 genes.^13^

We remain convinced that the Sugen-based models will continue to teach us, not just about the transition from the initial pulmonary endothelial cell damage (how does VEGF receptor blockade and/or activation of the AhR-cytochrome axis kill endothelial cells?) to exuberant proliferation of phenotypically altered cells (how do vascular cells become apoptosis-resistant?). The models can teach us about pulmonary intravascular inflammation and lung vascular immunity and right heart failure (how important is the impaired myocardial microcirculation?).

Beginning in 2000, when Peter Hirth at Sugen in South San Francisco provided the first batch of SU5416, until today, it has been an interesting journey. In our opinion, there are important questions that work with this model can answer. Here are just a few: Because the Sugen/hypoxia model is a “two hit model,” as are the subsequently developed Sugen-based rat models,^8^,^9^,^19^ it is critical for a deeper understanding of the pathogenesis to investigate how two hits interact on a cellular and molecular level. Of note, it is generally accepted that for human severe PAH to develop, also two hits are necessary and one of which can be genetic. The Sugen model of a genetically susceptible rat strain which develops severe PAH without hypoxia,^20^ could perhaps be a useful tool for such investigations.

While the PAH in Sugen/hypoxia model is unresponsive to conventional vasodilator treatments,^6^ fasudil injection in anesthetized rats with established severe PAH revealed that—in spite of widespread pulmonary vessel obliteration—the pulmonary artery pressure could be acutely decreased.^7^ The mechanism of this vaso-relaxing effect of fasudil is not understood. Investigation of the mechanism of action of this drug may lead to the discovery of new classes of pulmonary vasodilators.

Finally, it appears to us that intact signaling through the VEGF→Akt axis is critically important for the homeostasis of pulmonary vascular endothelial cells. In the Sugen/hypoxia model, treatment with Sugen5416 resulted in a significantly decreased expression of the genes encoding VEGF, VEGFR-2 and phospho Akt .Which are the feed-forward mechanisms that cause this effect?

Perhaps the next 20 years of investigating the Sugen models will provide some of the answers.
